# Electrochemical Affinity Biosensors: Pervasive Devices with Exciting Alliances and Horizons Ahead

**DOI:** 10.1021/acssensors.3c01172

**Published:** 2023-08-03

**Authors:** Susana Campuzano, José M. Pingarrón

**Affiliations:** Departamento de Química Analítica, Facultad de Ciencias Químicas, Universidad Complutense de Madrid, 28040 Madrid, España

**Keywords:** electrochemical affinity biosensors, wearable, microfluidics, precision medicine and nutrition, artificial intelligence

## Abstract

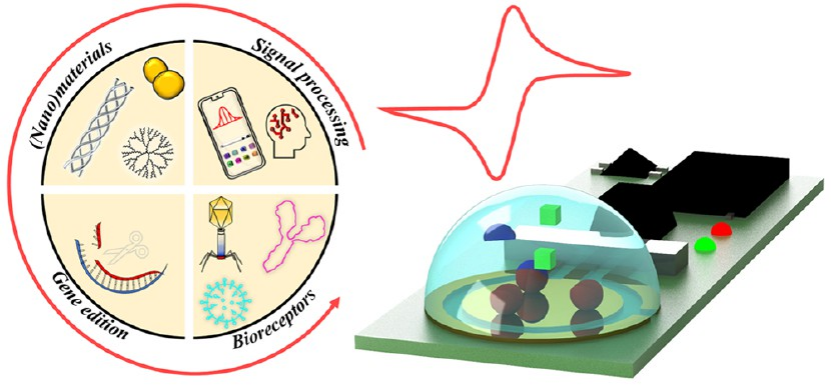

Electrochemical affinity biosensors are evolving at breakneck speed, strengthening and colonizing more and more niches and drawing unimaginable roadmaps that increasingly make them protagonists of our daily lives. They achieve this by combining their intrinsic attributes with those acquired by leveraging the significant advances that occurred in (nano)materials technology, bio(nano)materials and nature-inspired receptors, gene editing and amplification technologies, and signal detection and processing techniques. The aim of this Perspective is to provide, with the support of recent representative and illustrative literature, an updated and critical view of the repertoire of opportunities, innovations, and applications offered by electrochemical affinity biosensors fueled by the key alliances indicated. In addition, the imminent challenges that these biodevices must face and the new directions in which they are envisioned as key players are discussed.

Currently, the monitoring of molecules is the focus of research objectives at a global level due to the recognized and unique potential to improve our quality of life by monitoring the environment or ‘measuring’ the states of health.^1^

Although ELISA and PCR technologies are considered the gold standards for the determination of proteins and nucleic acids, respectively, electrochemical biosensors involving affinity reactions are establishing as complementary and/or alternative tools. They match widely accepted technologies in characteristics such as sensitivity and selectivity. But, in addition, due to their portability and the functional simplicity of the technology, electrochemical affinity biosensors offer a more competitive avenue for rapid analysis and for application in portable and miniaturized point-of-need (PON) devices with desirable cost-effectiveness, thus allowing democratic and broad accessibility of precision information to individuals.^1−3^ It is important to note that the relentless stream of advances and capabilities that electrochemical affinity biosensors have acquired through unique partnerships makes their scope ever broader. The latest developments invite us to think that we have underestimated their potential and that they offer a very promising horizon not only for the competitive determination, in terms of simplicity, cost, and PON, of molecules already identified and validated by other technologies but also for the discovery and validation of molecular markers in different fields of interest: clinical, therapeutic, nutritional, environmental, safety, etc.

Indeed, the tremendous progress that electrochemical biosensors, particularly those involving natural and biomimetic (excluding molecular imprinted polymers, MIPs) affinity receptors, have experienced in recent years along with their impressive versatility and adaptability to couple other materials and technologies^4^ have made that they are considered ubiquitous devices for studies at the molecular level and for the ability to track molecules providing quantitative information in a simple and quick manner and in any environment. This kind of information is essential for advancing precision medicine, therapy, and nutrition, with the enormous benefits that this represents at both the individual and societal levels.

In light of the above, this perspective article, which can be considered of broad interest to the scientific community due to the increasing incursion of these devices in different fields and their unstoppable alliances with more and more technologies and disciplines, aims to provide the reader, a general, updated, and critical view, supported by recent representative and illustrative literature, of the repertoire of opportunities, innovations, and applications offered by electrochemical biosensors based on natural and biomimetic (excluding MIPs that would merit a separate review article) affinity receptors.

This perspective is structured in three main sections. First, the intrinsic capabilities of these devices are discussed. The second section provides a panoramic and updated view of the routes covered by these devices through decisive unique partnerships, grouped into four categories: (nano)fabrication and materials technology; bio(nano)materials and nature-inspired receptors; gene editing and amplification technologies; and signal detection and processing techniques. Finally, the third section offers a somewhat more personal perspective on the most imminent challenges and new envisaged directions.

## Devices with Unique Intrinsic and/or Acquired Attributes

Currently, electrochemical affinity biosensors exhibit a large battery of attractive features both of intrinsic nature or acquired by unique alliances with other materials, strategies, and technologies.

The special characteristics of the electrochemical substrates, instrumentation, and techniques endow electrochemical biosensors with *intrinsic features* that include affordability, low-power requirement, portability, sensitivity, specificity, timeliness, ease of use, feasibility for analyzing turbid, opaque, and colored solutions, and easy integration with PON technologies. These features make electrochemical biosensors ideal to be employed by anyone in remote or resource-limited settings.^1,5,6^ Currently, electrochemical substrates are manufactured cheaply and on a large scale, while electrochemical instruments are commercially available in sizes so small that we could never imagine but keeping a performance equivalent to traditional instruments with the size of a microwave oven.^1^

**Figure 1:**
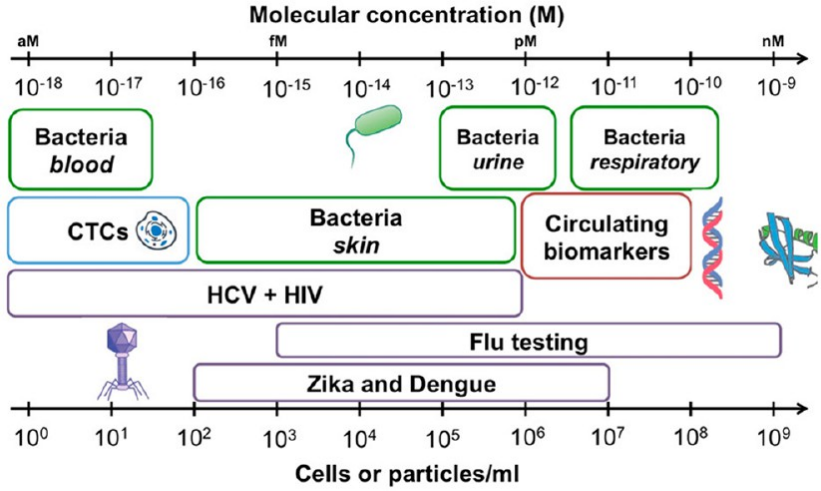
Ranges of clinical interest for different molecular markers. Reprinted with permission.^7^ Copyright 2017, American Chemical Society.

Electrochemical biosensors have rapidly profited from the innovations that other technologies have undergone to empower themselves with unique *acquired attributes and capabilities*. These devices can currently boast, among many other things, of:

- Being sustainable, edible, biocompatible and/or regenerative.
- Performing multiplexed determinations even at different molecular levels.
- Combining in the same device various bioassay formats and enzymatic markers as well as different detection modes allowing the development of multimodal biotools.
- Performing their work outside the workbench and even in the body and autonomously in devices that we ingest, wear (tattoo, microneedle-based, watch, contact lens, etc.), or have implanted.
- Tracking clinically relevant levels (Figure 1) of a wide variety of markers (proteins, antibodies, glycoproteins, cytokines, hormones, proteases, ion channels, point mutations, lnRNAs, mRNAs, methylated DNAs and RNAs, cells, secretomes, exosomes, and more...) directly in body fluids (extracted, circulated, or secreted either naturally or stimulated) such as serum, urine, blood,^3^ plasma, saliva, interstitial fluid, tears, and sweat, often addressing the “needle in a haystack” challenge, and for targets outnumbered by a million-fold excess of nontarget species.^7,8^

- Being able to cope with the undesirable and dreaded nonspecific binding in physiologically relevant environments^9−11^ by modifying the electrochemical devices with antibiofouling and/or protective properties allowing working in highly complex or denaturing media.^2^
- Allowing the reagentless analysis of markers of different molecular level (nucleic acids, proteins, bacteria, viruses, drugs) in real-time in discrete or continuous mode.^12^
- Operating without calibration and/or washing steps.^13^
- Monitoring the same biomarkers in both liquid and needle biopsies, thereby providing information on both primary and circulating disease.^14^
- Allowing the robust identification of single nucleotide polymorphisms (SNPs) using a few μL fingerpick sample.^15^
- Shedding light on the genome, the transcriptome, the proteome, the glycome, the microbiome,^16^ and the importance and complexity of the humoral immune response and epigenetic memory.^17^

## Key Alliances to Cover Important Routes

Due to the ubiquitous and open characteristics of electrochemical affinity biosensors, a breadth of opportunities and applications has been recently reported to venture into many different specific disciplines addressing highly relevant and challenging issues of broad interest to both the scientific and social communities.

**Figure 2:**
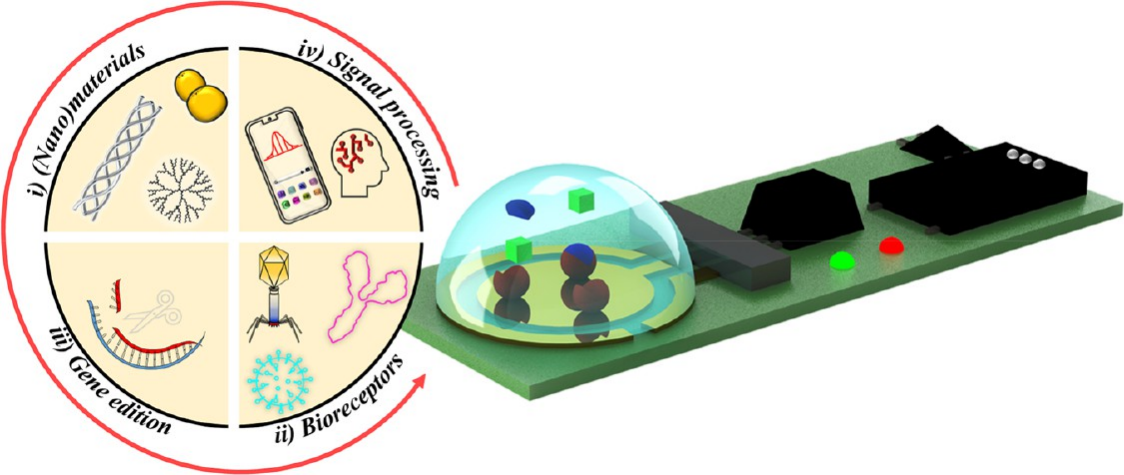
Modern electrochemical affinity biosensors provide important advances in (i) (nano)materials manufacturing and technology; (ii) bio(nano)materials and nature-inspired receptors; (iii) gene editing and amplification technologies; and (iv) signal detection and processing techniques. Figure drawn by Eloy Povedano and Víctor Ruiz-Valdepeñas Montiel, members of the research team of the authors of this perspective article.

Indeed, the meteoric progress that electrochemical affinity biosensors have experienced in recent years mostly stems from the intelligent exploitation of important advances in other fields (Figure 2). These key alliances can be grouped into four broad categories: (i) (nano)materials manufacturing and technology; (ii) bio(nano)materials and nature-inspired receptors; (iii) gene editing and amplification technologies; and (iv) signal detection and processing techniques. Such disciplines have been instrumental in overcoming the challenges for technologies to contribute to the research and future implementation of precision medicine, therapy, and nutrition, where many of society’s current demands can be framed. Therefore, the potential of each of these alliances to address particularly relevant and often disruptive challenges is critically discussed below in the light of representative examples selected from the recent literature.

### (Nano)materials Manufacturing and Technology

**Figure 3:**
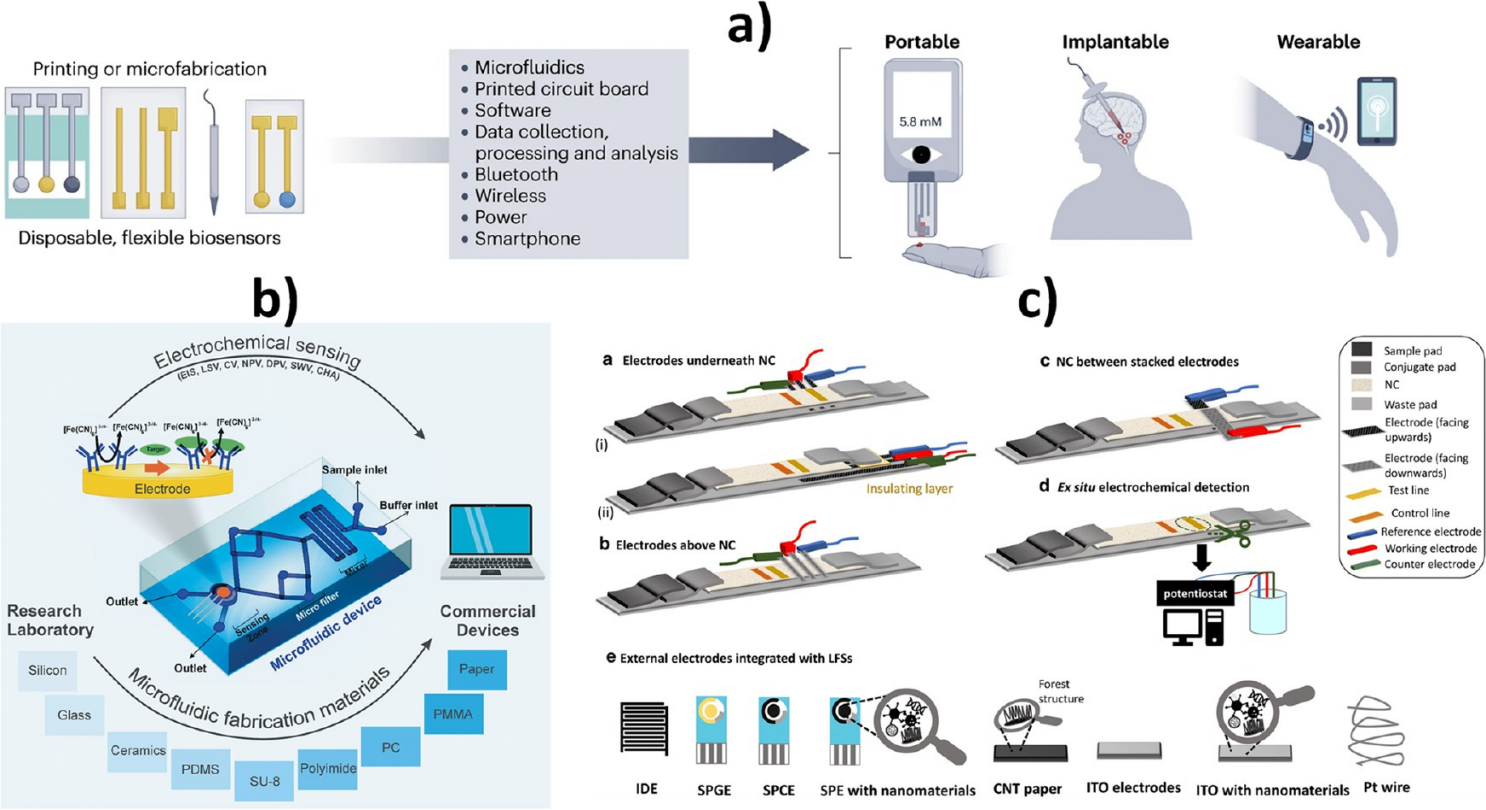
(a) Electrochemical biosensors integrated in portable, wearable, and implantable devices. (b) Schematic representation of electrochemical methods and microfluidic fabrication materials for the design of microfluidic electrochemical devices. (c) Illustrative diagram of existing principles for integrating electrodes into lateral flow strips (LFS) and electrode types and modifications used in developing eLFAs. (a) Reproduced with permission.^31^ Copyright 2023, Nature Publishing Group. (b) Reprinted with permission.^25^ Copyright 2022, Springer. (c) Reprinted with permission.^26^ Copyright 2021, Springer.

Innovations in materials science, microfabrication, flexible microelectronic and microfluidics technology, the advances in the design and fabrication of electrochemical substrates, as well as the progress in miniaturized and flexible bioelectronics, and in different apps^18^ have enabled the development of electrochemical biosensors in new formats: ingestible,^19^ wearable,^20^ and implantable that allow the sensors to be carried on the own body or daily life devices (smartphones/watches) (Figure 3a). The versatility of current electrochemical substrates in terms of use (reusable or disposable^21,22^), design, fabrication materials (paper,^23^ sustainable, edible,^24^ plastic, textile, and polymeric), and properties (superwettable, flexible, and stretchable) should be highlighted. Electrochemical affinity bioassays have also been successfully integrated into microfluidic^25^ (Figure 3b) and lateral flow (eLFA) devices (Figure 3c).^26,27^

Moreover, advances in microfabrication and microfluidic tecnologies^28^ have led to the development of integrated devices able to unify the sample collection, preparation, analysis, and postprocessing (known as “sample-in-answer-out” type of devices^29^) or able to obtain multiplexed^30,31^ and/or multiomic information in a minimally invasive manner.

Moreover, the development and integration of single or hybrid artificial nanomaterials (these latter endowed with synergistic effect) has led to the development of devices with antifouling properties and improved sensitivity, selectivity, robustness, and/or stability. Intrinsic properties of these nanomaterials, such as fast electron transfer, biocompatibility, and electrocatalytic and pseudoenzymatic activity, have been exploited in electrochemical affinity biosensors by using them as electrode modifiers, advanced labels, nanotransporters of signaling elements, and enzyme mimickers.^32^

**Figure 4:**
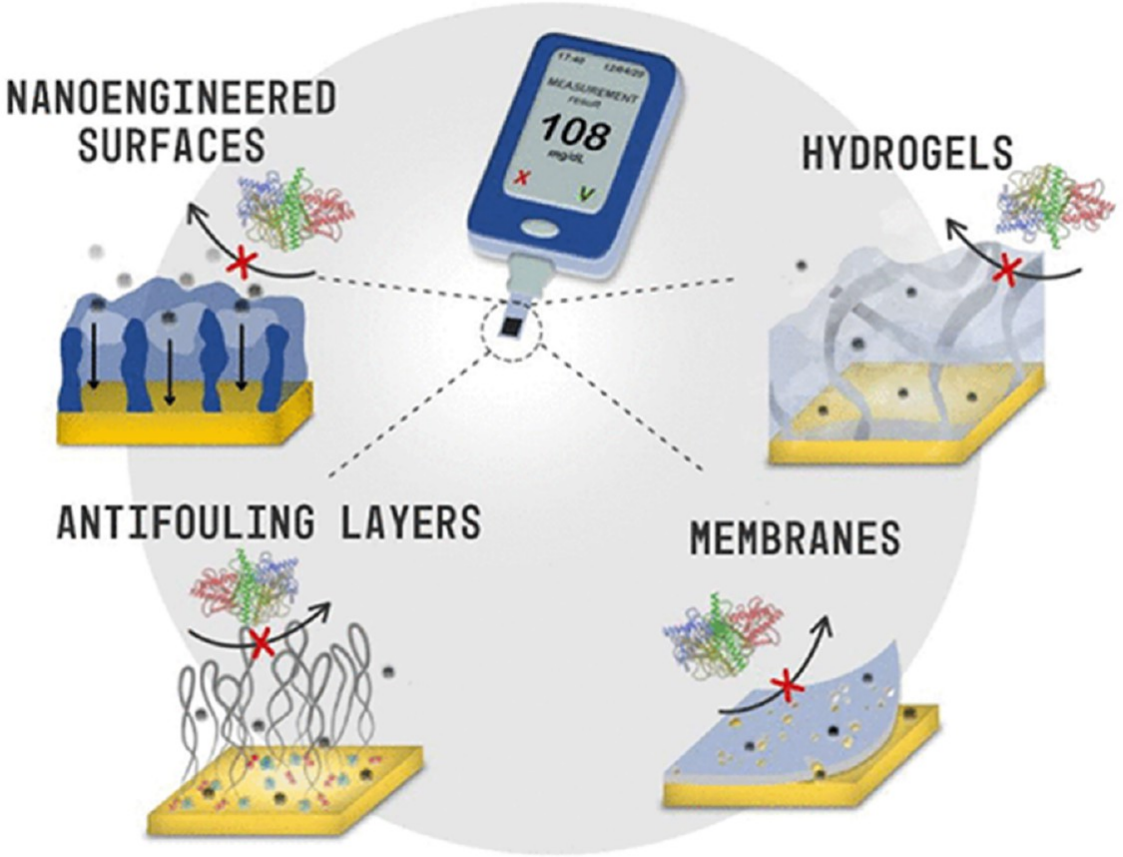
Antifouling/protective strategies for electrochemical biosensing. Reprinted with permission.^2^ Copyright 2021, American Chemical Society.

The use of nanoengineered surfaces (i.e., nanoporous metals and nanocarbons), antifouling layers (PEG, polymers,^33^ and peptides^34^), nanoporous membranes,^35^ mesoporous films,^36,37^ and hydrogels^2^ has imparted electrochemical affinity biosensors attractive antifouling and/or protective properties, allowing addressing discrete or continuous determinations directly in very complex and/or denaturing matrices (Figure 4).

The use of smart “dispersible electrodes”, based on the use of electrically reconfigurable networks of Au-coated magnetic nanoparticles (Au@MNPs), that ingeniously combine electrical conductivity and magnetic properties, modified with the appropriate receptor to selectively recognize the analyte, has led to bioplatforms that allow ultrasensitive (in the aM range) and rapid (20–30 min) determination of a wide variety of relevant targets (miRNAs,^38^ circulating tumor DNA,^39^ and point mutations in relevant genes^40^) directly in raw biofluids.

**Figure 5:**
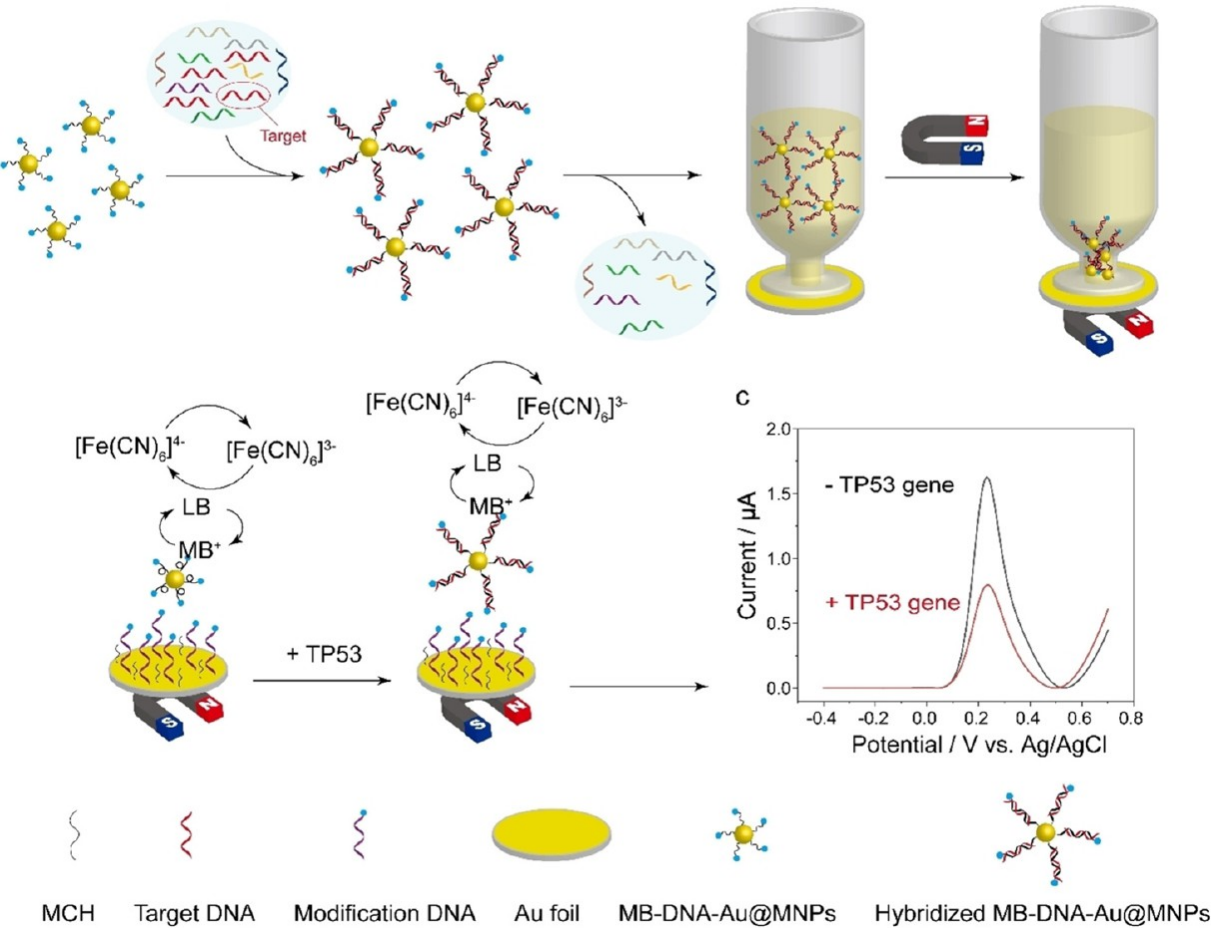
Schematic display of a “dispersible electrode” for the detection of the *TP53* gene mutation in blood. Reprinted with permission.^40^ Copyright 2022, Wiley-VCH.

As an example, Figure 5 shows a “dispersible electrode” for the determination of point mutations in the tumor suppressor *TP53* gene.^40^ This method combined the use of Au@MNPs with a DNA probe, complementary to the target DNA, double-labeled with thiol and methylene blue (MB), with the use of a gold macroelectrode modified with a binary monolayer of a DNA probe (also labeled at both ends with thiol and MB) and mercaptohexanol (MCH). As can be seen, the MB-DNA-Au@MNPs were exposed to the target DNA with which they hybridized and were captured with a magnet on the surface of the gold electrode. The decrease in the MB oxidation voltammetric signal observed in the presence of the target DNA was attributed to the increased distance between the nanoparticles after hybridization, which hindered electron transport through the network.

**Figure 6:**
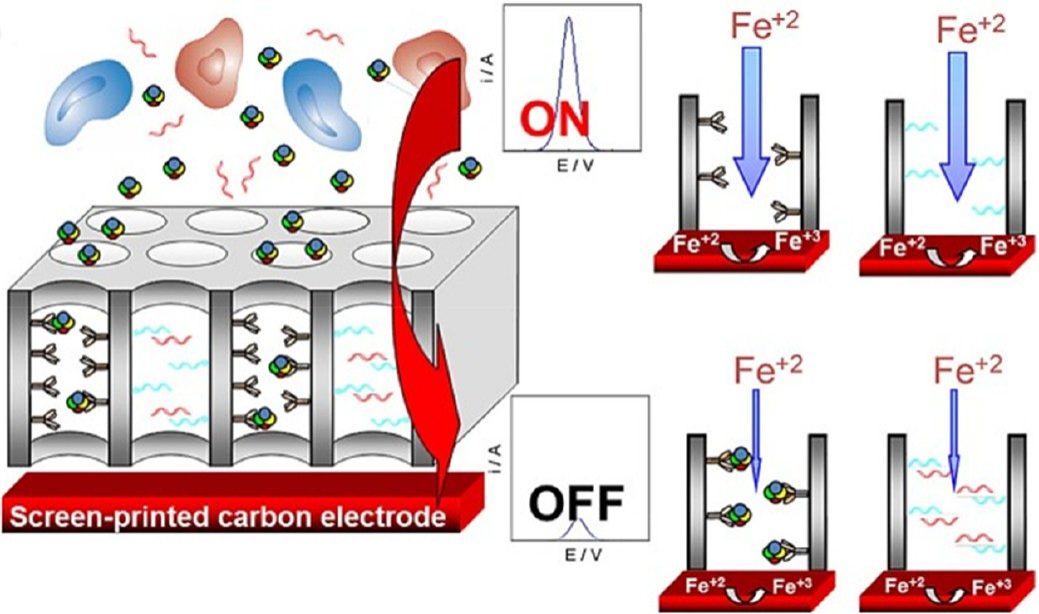
Principles of voltammetric affinity biosensing at electrodes modified with nanoporous membranes functionalized with specific bioreceptors in the absence (“On” response) and presence (“Off” response) of the target biomolecule. Reproduced with permission.^43^ Copyright 2016, Elsevier.

The use of nanoporous membranes as electrode modifiers has been exploited to develop attractive electrochemical affinity biosensing strategies to detect markers of different nature in a label-free, simple, sensitive, and less prone to matrix interference way.^41−45^ These strategies take advantage of the analytical response blocking or unblocking (decrease or increase of the electrochemical response of a redox marker) produced after the analyte recognition process on the biofunctionalized membrane. As an example, Figure 6 shows the schematic representation of a biosensing method by measuring the voltammetric signal decrease of a redox marker (K_3_[Fe(CN)_6_]) due to its diffusion blocking to the electrode surface through nanochannels modified with a specific antibody or nucleic acid upon the affinity reaction. In these methods, nanoporous membranes functioned both as sensing platforms and as filters of big-sized interferences in real samples, allowing minimization of matrix effects. Both the size and thickness of the nanopore played a determining role in the biosensing strategy sensitivity.

**Figure 7:**
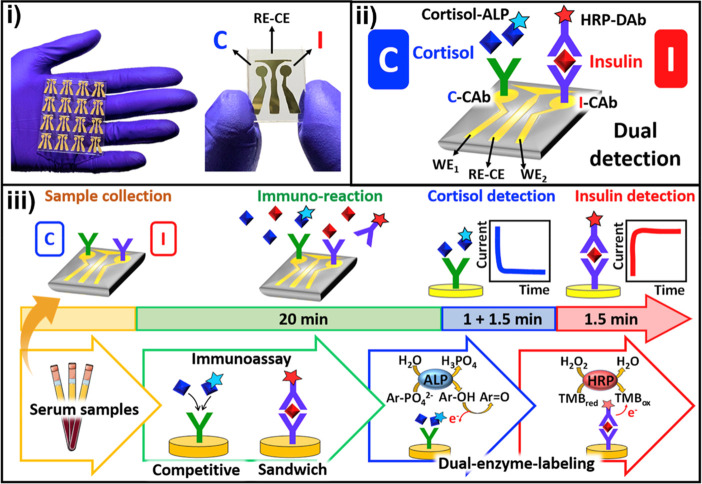
Biosensor chip integrating immunoassays involving different formats and enzymatic tracers for the simultaneous determination of cortisol and insulin. Reprinted with permission.^47^ Copyright 2020, Elsevier.

Disposable electrochemical chips that combine catalytic and affinity bioassays or immunoassays with different formats and enzyme tracers in the same device have been developed. These chips allow simultaneous on-the-spot determination of important diabetes biomarkers (glucose and insulin^46^ or cortisol and insulin,^47^Figure 7) using a single 10 μL droplet of blood serum in less than 30 min. These capabilities make them very attractive for improving the management of diabetes by performing minimally invasive and rapid screening in decentralized settings.

Advances in microfabrication and microfluidics technologies have been harnessed in the development of microfluidic electrochemical biodevices as well as of organs-on-chips (OoCs) and LFAs devices.

Multiplexed electrochemical microfluidic biosensors have been developed for on-site measurement of multiple analytes from one sample and/or of the same analyte from different samples simultaneously, thus enhancing the accuracy of the diagnosis of diseases and their therapy success.^25,30,48^

Electrochemical affinity biosensors have been also employed for determining cell viability, differentiation and function in 2D cultures, and, more recently, in 3D cultures (organoids, spheroids, and OoCs) more reflective to mimic the real 3D physiological conditions.^49^ These are essential aspects for the application of these biosensors as tools in pharmaceutical analysis and toxicity testing.^50^ An illustrative example is the universal label-free, impedimetric transduction-based platform integrating electrochemical affinity biosensors (employing aptamers or antibodies as capture bioreceptors) on microfluidic chips.^51^ This platform, which enabled online functionalization of microelectrodes and regeneration of sensors, was applied to the online and nanomolar-range determination of a wide variety of biomarkers and was able to be directly connected to OoC systems.

**Figure 8:**
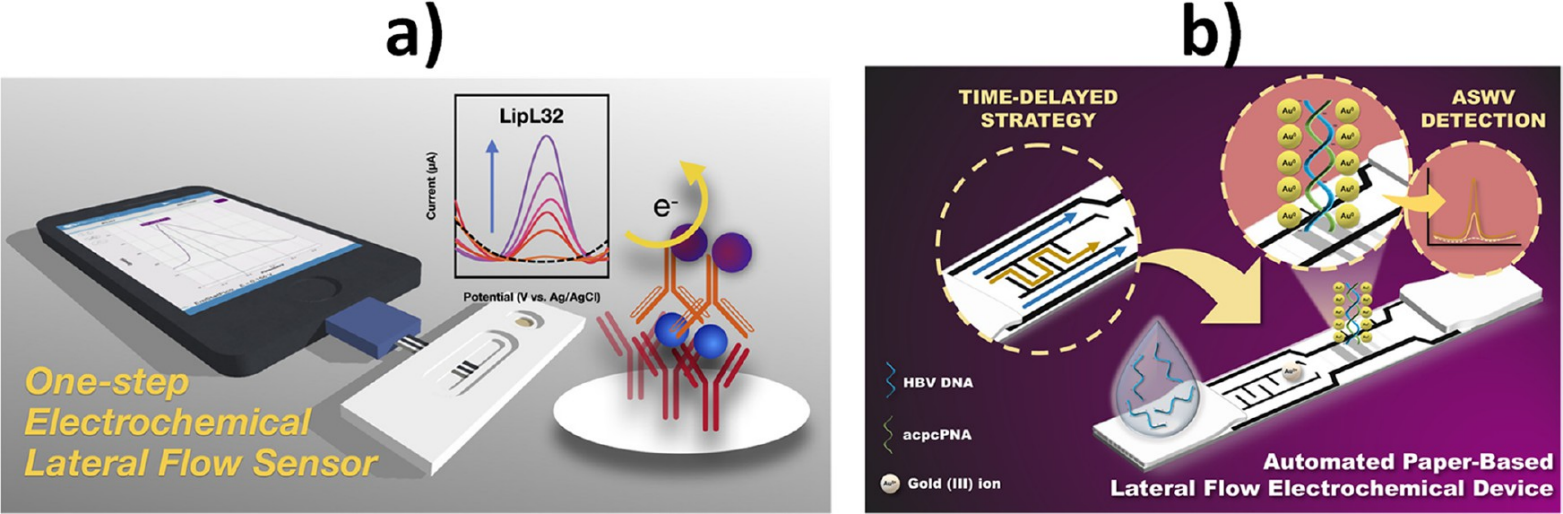
Schemes of eLFAs for the determination of protein (a) and genetic (b) biomarkers. (a) Reprinted with permission.^54^ Copyright 2022, American Chemical Society. (b) Reprinted with permission.^56^ Copyright 2021, American Chemical Society.

eLFA devices continue to make steady progress and, in addition to the determination of relevant protein markers (Figure 8a),^52−55^ have been proposed recently for the quantitative determination of nucleic acids. The paper-based eLFA device shown in Figure 8b was successfully applied for the determination of hepatitis B virus DNA in patient sera without any amplification step and with a total operation time of 7 min.^56^

### Bio(nano)materials and Natural-Inspired Receptors

The use of multimeric or multifunctional probes involving peptides and nucleic acids and bioreceptors inspired by nature has endowed electrochemical affinity biosensors with important capabilities and has significantly expanded their scope far beyond the most commonly pursued objective, such as the determination of biomarkers. Indeed, they are suitable for the identification of new markers and testing their clinical potential.

The versatility of use along with the unique features provided by biological (nano)materials (peptide and nucleic acid nanomaterials, e.g., nanotweezers, nanospheres, and “Y-shaped”, tetrahedral, origami, and dendrimer nanostructures) have led to the development of electrochemical affinity biosensors with empowering properties in terms of sensitivity, storage stability, assay time, simplicity, and robustness, currently considering the exit of these devices from the research laboratory to the real world as more feasible than unattainable.^57−61^

Like artificial nanomaterials, these bio(nano)materials can be exploited in electrochemical biosensors, as electrode modifiers, signaling elements, and/or their transporters. But unlike the artificial ones, bio(nano)materials can also be used as biorecognition elements and, in the case of peptides, as substrates to determine proteins with specific activities, such as proteases for which the peptide serves as the cleavage-sensing element.

DNA is inherently an excellent self-assembly (nano)material due to its predictable base pairing, high chemical stability, and convenience of synthesis and modification. These unique properties explain its increasing use in electrochemical biosensors; as a recognition element; to generate unique assemblies using “Y-shaped”, tetrahedral, origami, and dendrimer nanostructures, nanospheres, networks, and hydrogels; and as a multifunctional biomaterial using multifunctional probes.^61^

“Y-shaped” DNA nanostructures are generally formed by the partial hybridization of two or three oligonucleotides. The use of such nanostructures allows the probe to be stably attached to the electrode surface, to remain upright with proper spacing, and to offer higher recognition efficiency compared to conventional single-stranded probes, especially for long targets, also minimizing nonspecific adsorptions.^62−64^

This type of nanostructure has been exploited, alone or coupled to other amplification strategies, in the development of biosensors for the determination of relevant nucleic acids, achieving LODs at fM levels for the determination of DNAs and short RNAs^65^ and at pM levels for the determination of long RNAs.^66^ They have been successfully applied to the analysis of highly complex samples (serum, total cellular RNA, exosomes, and urine).

The use of tetrahedral and origami DNA nanostructures provides an effective alternative to improve the recognition capabilities of DNA probes confined on electrode surfaces because they allow control of the surface chemistry, conformation, packing density and spacing of the immobilized recognition probe, and the size and geometry of the formed nanostructures.^67^

The tetrahedral DNA nanostructures are generated simply, rapidly, and reproducibly through single-step self-assembly of four probes (three of them thiolated) on gold surfaces without the need to use MCH to lift the capture probe and minimize nonspecific adsorptions. They exhibit excellent mechanical rigidity and structural stability, which confer robustness and reliability to the detection process. It is important to note that surfaces modified with such nanostructures are fully compatible with electrochemical detection. Although they form a relatively thick layer (∼6 nm), the hollow structure of these nanostructures facilitates the diffusion of the electroactive species to the electrode surface.^61,67^

The use of tetrahedral DNA nanostructures as electrode modifiers allows the control of the spacing and density of the immobilized probe, thus improving the stability of the biosensor (three anchor points versus the single point of single-stranded recognition probes), the accessibility of the target, and the ability to provide the biodevice with antifouling properties.^61,67,68^

These nanostructures have also been useful to amplify the electrochemical response when employed as transporters of multiple signaling elements by taking advantage of their four vertices.^69^

Electrochemical biosensors using these DNA nanostructures have been employed for the determination of a wide variety of molecules. Some of these DNA nanostructures are combined with the use of artificial nanomaterials, DNAzymes, and other amplification strategies to achieve sensitivities at the aM-fM level for nucleic acids and fM for proteins.^61^

In recent years, origami DNA nanostructures have attracted great interest due to their large surface area and unprecedented customization potential to precisely arrange target recognition sites on the nanoscale. Like tetrahedral nanostructures, origami nanostructures allow the immobilization of multiple single-stranded DNA probes at predetermined and density-controlled positions, thus improving their accessibility and recognition efficiency. However, they do not involve thiol chemistry.^70,71^ Despite this, due to their larger surface area, they allow the immobilization of a larger number of probe molecules than tetrahedral nanostructures. Nevertheless, to date, origami DNA nanostructures have been scarcely explored in electrochemical biosensors, which can be attributed to the lack of maturity of the tools for their design.

DNA dendrimers, highly branched nanostructures formed by sequential complementary hybridization of pre-engineered DNA components to simply and stably anchor many signaling elements (small molecules, biomolecules, or metal nanoparticles) have been employed as carriers of signaling elements to amplify the electrochemical response, improving the sensitivity and extending the linearity range of the resulting electrochemical biosensors.^72,73^

Although much less exploited than other DNA nanostructures, nanospheres have also been employed in electrochemical biosensors to preconcentrate large amounts of signal indicators from the solution to the vicinity of the electrode surface, accelerating and favoring electronic transfer.^74^

DNA hydrogels and networks have also shown potential for improving the sensitivity of electrochemical biosensors. DNA networks have been used also to reduce the assay time by employing the concept of “dispersible electrodes” pioneered by Prof. Gooding’s research group^38,39^ and for amplification purposes coupled to more conventional electrochemical biosensors mostly involving electrochemical impedance spectroscopy (EIS) detection. In this latter case, DNA networks generated by self-assembly of DNA probes doubly functionalized with biotin in the presence of streptavidin^75^ or by isothermal nonlinear chain hybridization strategies^76^ have been employed.

DNA hydrogels are three-dimensional porous network polymers containing a large amount of water that are constructed by cross-linking only nucleic acids (pure hydrogels) or by grafting DNA strands onto hydrophilic polymers or other materials (hybrid hydrogels). Pure hydrogels have more limited mechanical properties and higher cost than hybrid hydrogels, and, therefore, hybrid hydrogels have been the most widely used to date in electrochemical biosensors.^61^

Although not yet widely explored, DNA hydrogels are considered promising biomaterials in electrochemical biosensors because of their biocompatibility, flexibility, large specific surface area and loading capacity, rapid diffusion of small molecules, mechanical stability, and responsiveness to appropriate stimuli (such as pH, light, etc.).

These biomaterials have been used as three-dimensional electrode modifiers, to trap catalytic or affinity receptors or to transport electrons and to amplify the electrochemical response.^61^

An illustrative example is the biosensor constructed for the determination of miRNAs by immobilizing a hybrid hydrogel on an indium tin oxide/polyethylene terephthalate (ITO/PET) electrode.^77^ Ferrocene (Fc)-labeled recognition probes were grafted onto a polyacrylamide polymer, and their hybridization with the target miRNA led to dissolution of the hydrogel and the decrease of the Fc oxidation current monitored by differential pulse voltammetry.

The generation of hydrogels on the electrode surface has also been exploited for amplification purposes in the development of impedimetric biosensors.^78^ Importantly, the possibility of stimulating the hydrogel density by pH to modulate signal amplification was demonstrated in these devices.

The use of multifunctional DNA probes, i.e., those capable of performing other functions in addition to target recognition, is particularly noteworthy for the development of cutting-edge electrochemical bioplatforms. Among these multifunctional DNA probes, polyA-type probes (comprising a polyA tail and a recognition part) and DNAzymes (single-stranded nucleic acids capable of catalyzing a specific chemical reaction in the presence of a specific target),^61^ are noteworthy.

**Figure 9:**
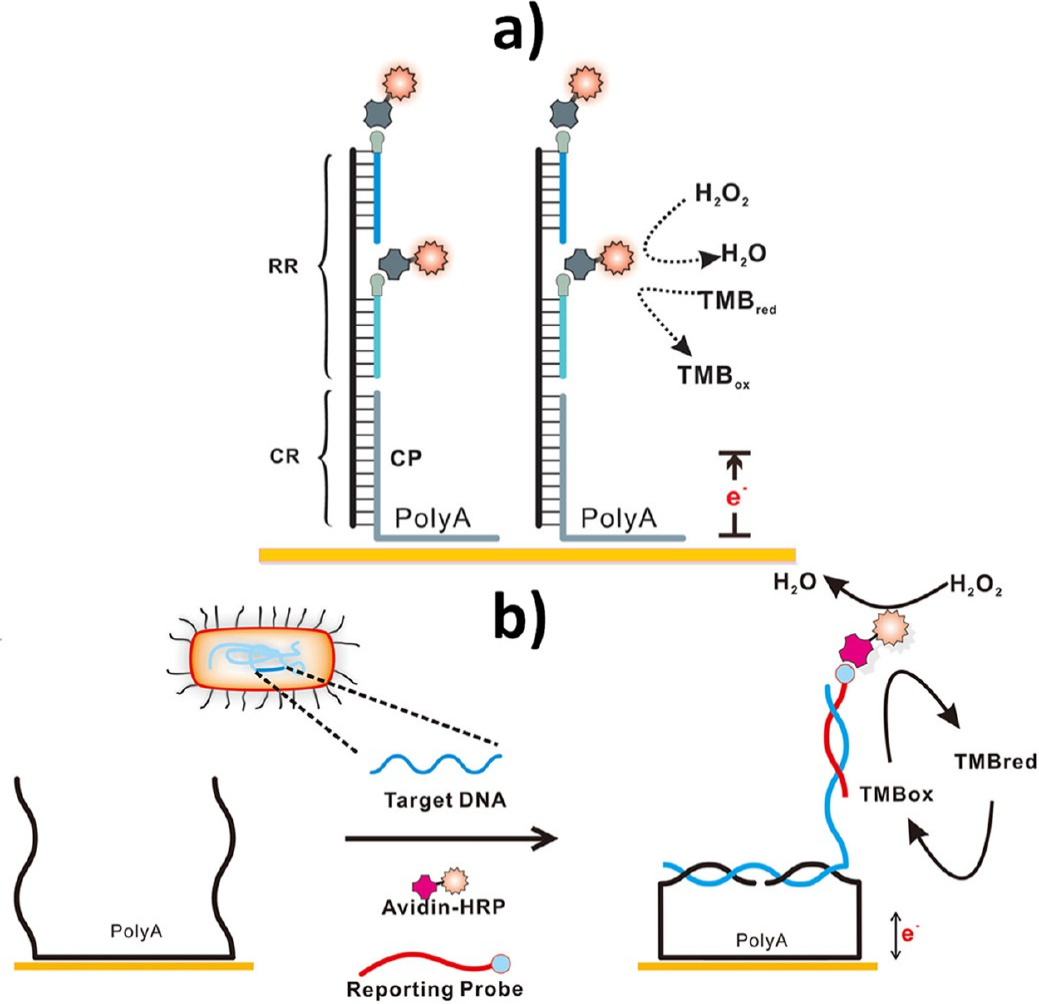
Examples of electrochemical affinity biosensors based on the use of single (a) or multiblock (b) polyA capture probes for the determination of bacterial genetic material. (a) Reproduced with permission.^81^ Copyright 2019, American Chemical Society. (b) Reproduced with permission.^82^ Copyright 2019, American Chemical Society.

PolyA probes adsorb across adenines strongly to gold electrode surfaces with an affinity comparable to that of the Au–S chemical bond. Similar to thiolated ternary monolayers, composed of a thiolated probe, a dithiol (cyclic or linear), and MCH,^79,80^ polyA probe monolayers allow controlling the capture probe density and spacing to achieve optimal hybridization efficiency, minimize nonspecific adsorptions, and impart antifouling properties to the modified surface.^61,81,82^ However, unlike thiolated ternary monolayers, polyA probe monolayers are monocomponent, more economical, do not involve thiol chemistry, and exhibit increased storage stability due to the larger number of probe anchor points to the gold surface. In addition, the use of polyA probes allows modulation at will of the polyA fragments’ size as well as the evaluation of their influence on hybridization efficiency, sensitivity, and other interesting properties of the bioplatform such as storage stability and antifouling capacity. Bioplatforms based on the use of single (Figure 9a) or multiblock (in which two recognition sequences are connected through a polyA fragment, Figure 9b) polyA capture probes have been proposed for the determination of nucleic acids at the fM level with no need for any other amplification strategy.

DNAzymes have been used for amplification purposes in the development of electrochemical biosensors for the sensitive determination of analytes of different nature (metals, viruses, bacteria, and others).^83^ A representative example is an electrochemical affinity biosensor for the detection of bacterial urinary tract infections in less than 1 h by using DNAzymes dually functionalized with biotin and MB. The DNAzymes were designed to release an electroactive DNA in the presence of the bacterial target (*Escherichia coli*) and integrated into a two-channel electrochemical chip with nanostructured star-shaped electrodes near each other.^84^ The method utilized a differential electrochemical signal readout between the two channels: a release channel where the reporter DNA is lost in the presence of the target bacterium and, consequently, the electrochemical response decreased and a capture channel where the MB response increased.

Aptamers are defined sequences of single-stranded RNA or DNA between 70 and 100 nucleotides in size capable of specific, high-affinity recognition of their target molecule by three-dimensional folding of their strand. Aptamers are selected in vitro through oligonucleotide libraries using the SELEX method, and they continue to gain prominence in the development of electrochemical bioplatforms due to the advantages they exhibit over the most widely used affinity receptors, antibodies, such as their considerably smaller size; high chemical and thermal stability; reversible denaturation capacity; and rapid, simple, inexpensive, and scalable synthesis and modification without the need for cell culture or animal handling, which ensures consistent results with different productions.^85^ Furthermore, aptamers can be generated against a broad spectrum of target molecules, and in the case of the target species poorly soluble in aqueous media, they can be selected in deep eutectic solvents (DES) which are considered sustainable solvents. Aptamers have been used both as recognition elements and for amplification purposes in electrochemical bioplatforms.^86^

To improve the capabilities currently exhibited with electrochemical biosensing, a recent trend is the design and application of multifunctional and multimeric aptamers. The former include polyA aptameric probes and aptazymes, which can be designed even for the simultaneous determination of analytes of different nature and behave similarly to their DNA analogues and the latter, through the ligation of two or more binding domains, provide an improved affinity toward the target.^61^ Thus, for example, electrochemical bioplatforms using multimeric aptamers have been designed for the simple and rapid detection of SARS-CoV-2 spicule proteins from the original Wuhan strain and its variants of concern.^87^ Multimeric or multifunctional peptides have been used as bioreceptors^34^ or as electrode modifiers^88^ to prepare scaffolds that, in addition to endowing the modified surface with antifouling properties, improved the sensitivity of the resulting biosensor by ensuring optimal spacing between the immobilized bioreceptors for biorecognition.

Modern peptides are, like aptamers, experiencing an unstoppable boom in the development of high-performance electrochemical biosensors.^89,90^ Peptides are sequences of different lengths composed of synthetic or natural amino acids connected by peptide bonds. Their use as affinity bioreceptors in electrochemical biosensing is advantageous in terms of stability, selectivity, structural and sequence diversity, and biocompatibility.

Synthetic peptides can be easily obtained in high yield and, while retaining their affinity toward targets, can be modified with specific functional groups for immobilization or signaling by automated chemical synthesis, avoiding the need for laborious in vivo procedures.

Like aptamers, peptides are competitive with antibodies in the preparation of electrochemical biosensors because of their smaller size, lower cost, and ease of chemical synthesis. Compared to nucleic acids, peptides have different acid–base behavior and possess several functional groups that can enhance interactions, and thus affinity, with the target analyte.

These peptides are employed in the development of electrochemical affinity biosensors as (i) signaling elements/transporters; (ii) biorecognition elements to interrogate a wide variety of analytes; (iii) enzyme substrates, playing an irreplaceable role in detecting the activity (or inhibition) of clinically relevant enzymes, such as proteases (selectively cleaving peptides) and kinases (catalyzing phosphorylation reactions in the presence of specific peptides); and (iv) electrode modifiers exploiting the ability to interact and self-assemble into highly ordered structures to prepare biocompatible surfaces and/or with antifouling properties or nanoarrays with good electronic properties that allow the immobilization of other bioreceptors in a proper arrangement.^89^ Due to their distinguished properties, multifunctional peptides and peptides produced by cutting-edge technologies should be highlighted.

Representative examples of biodevices with enhanced sensitivity and/or antibiofouling properties using multidomain (different domains with different functions or “all-in-one” peptides) and multimeric (more than one binding domain) peptides,^34^ as well as peptides acting as sensing bioreceptors and nanotransporters of signaling elements,^91^ have been recently reported.

Profiting from their ability to create scaffolds for the immobilization of conventional bioreceptors, multifunctional peptides have been designed with characteristic “Y-shaped” or branched shapes, which, in addition to simplifying the biosensor preparation, improved the biosensor performance compared to the linear peptide. Thus, for example, peptides with an “inverted Y″ shape have been reported to prepare scaffolds that provided antibiofouling properties to the modified surface and improved the sensitivity of the resulting biosensor for the detection of the COVID-19 N-gene by ensuring optimal spacing between the immobilized bioreceptors for biorecognition.^88^

Furthermore, the use of nature-inspired bioreceptors produced by modern technologies (HaloTag, Phage display, and directed mutation) has been crucial in recent years for electrochemical affinity biosensing to (i) explore new biorecognition elements independently of their commercial availability; (ii) discover and test the clinical potential of new biomarkers and molecular signatures; and (iii) develop competitive bioelectroanalytical tools helping the implementation of precision medicine, therapy, and nutrition. These nature-inspired receptors include, among others, natural cell membranes,^92^ molecular switches (DNAs, aptamers or peptides, dually modified with a linker for immobilization on the electrode substrate, and a redox-active reporter that reversibly change between at least two conformations in response to the specific binding of a molecular target), inverted molecular pendulums (double-stranded DNAs containing at its distal end an antibody that recognizes the target analyte),^92−95^ peptides, protein, viral antigens, and proteoforms (all of the different molecular forms in which the protein product of a single gene can be found^96^).

For instance, molecular switches or inverted pendulums have been employed to develop reagent-free electrochemical biosensing devices with near real-time response. Due to its selectivity and adaptability, inverted molecular pendulum technology^95^ is particularly attractive for the increasingly sought-after multiplexed determinations.

Recent electrochemical biotools have also profited from advances in proteomics and targeted mutation to participate directly and decisively in the discovery of new markers at different omics levels. In fact, the identification of new molecular signatures is essential to advance precision medicine for the treatment of prevalent diseases. In this exciting field, bioplatforms assisted by magnetic beads (MBs) modified with nature-inspired antigens identified by directed proteomics (circulating antigens,^97^ exosomal antigens,^98^ peptides,^99^ and proteoforms^100^) and produced by HaloTag and phage display technologies, have been pioneeringly proposed for the discovery, validation, and determination of new characteristic molecular signatures. Examples of these signatures comprise autoantibodies (antibodies that react with self-antigens) against proteins or peptides for the early diagnosis of colorectal cancer,^97,98,100^ and the preclinical identification of Alzheimer’s disease.^99^

It is important to point out that, in addition to their use as capture bioreceptors, peptides deployed in phages have been successfully exploited as tracers in the development of immunoassay strategies with noncompetitive formats,^101^ where phage particles carried peptides capable of selectively recognizing the target, or with competitive formats^102^ using phage particles carrying peptides that mimic the target (this is because they are called peptidomimetics).

It is precisely the smaller size of peptides compared to conventional antibodies that allows their use as detector bioreceptors to determine small molecules using noncompetitive formats (generally superior to competitive formats in selectivity, sensitivity, kinetics, and working range).

Phage display peptides can be labeled with multiple signaling molecules, such as inorganic crystals,^101^ or with enzyme-conjugated antibodies selective to the phage used for expression,^102^ to amplify the electrochemical response.

**Figure 10:**
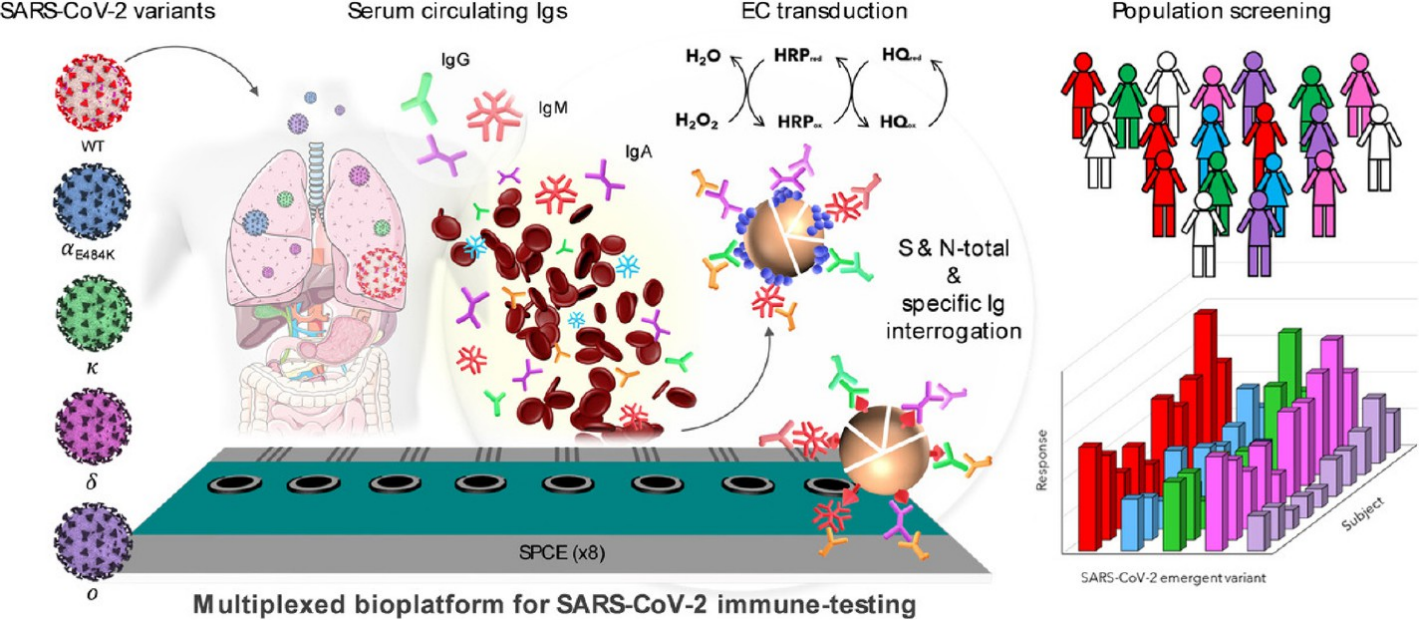
Bioplatform developed for the determination of serum levels of N- and S-specific total or individual immunoglobulin (Ig) isotypes (IgG, IgM, and IgA) by using MBs modified with N and in-house-expressed S ectodomains of SARS-CoV-2 variants. Amperometric detection at SPCEs. Reprinted with permission.^104^ Copyright 2022, Wiley-VCH.

Within the framework of the advances exhibited by electrochemical affinity biodevices for the detection of viral infections,^103^ and in particular for the management of the COVID-19 pandemic, a multiplexed and multipurpose bioplatform implementing indirect immunoassays on MBs modified with commercial nucleocapsid (N) protein or spike (S) protein ectodomains produced by targeted mutation, and using amperometric detection at screen-printed carbon electrodes (SPCEs), was reported for the determination of N- and S-specific circulating total or individual immunoglobulin (Ig) isotypes (IgG, IgM, and IgA) (Figure 10).^104^ The bioplatform allowed: (i) a reliable discrimination in 75 min between infected and noninfected patients using 1000-fold diluted sera; (ii) “quantification” of natural and/or acquired immunity following infection and/or vaccination processes, thus making possible the evaluation of the vaccination program’s efficacy and the implementation of personalized vaccination strategies in time and dose; (iii) evaluation of the humoral immune response against any variant that may arise; and (iv) identification of the variant responsible for the infection.^104^ Furthermore, the versatility of the bioplatform makes it easily transferable to the detection of other viral infections.

**Figure 11:**
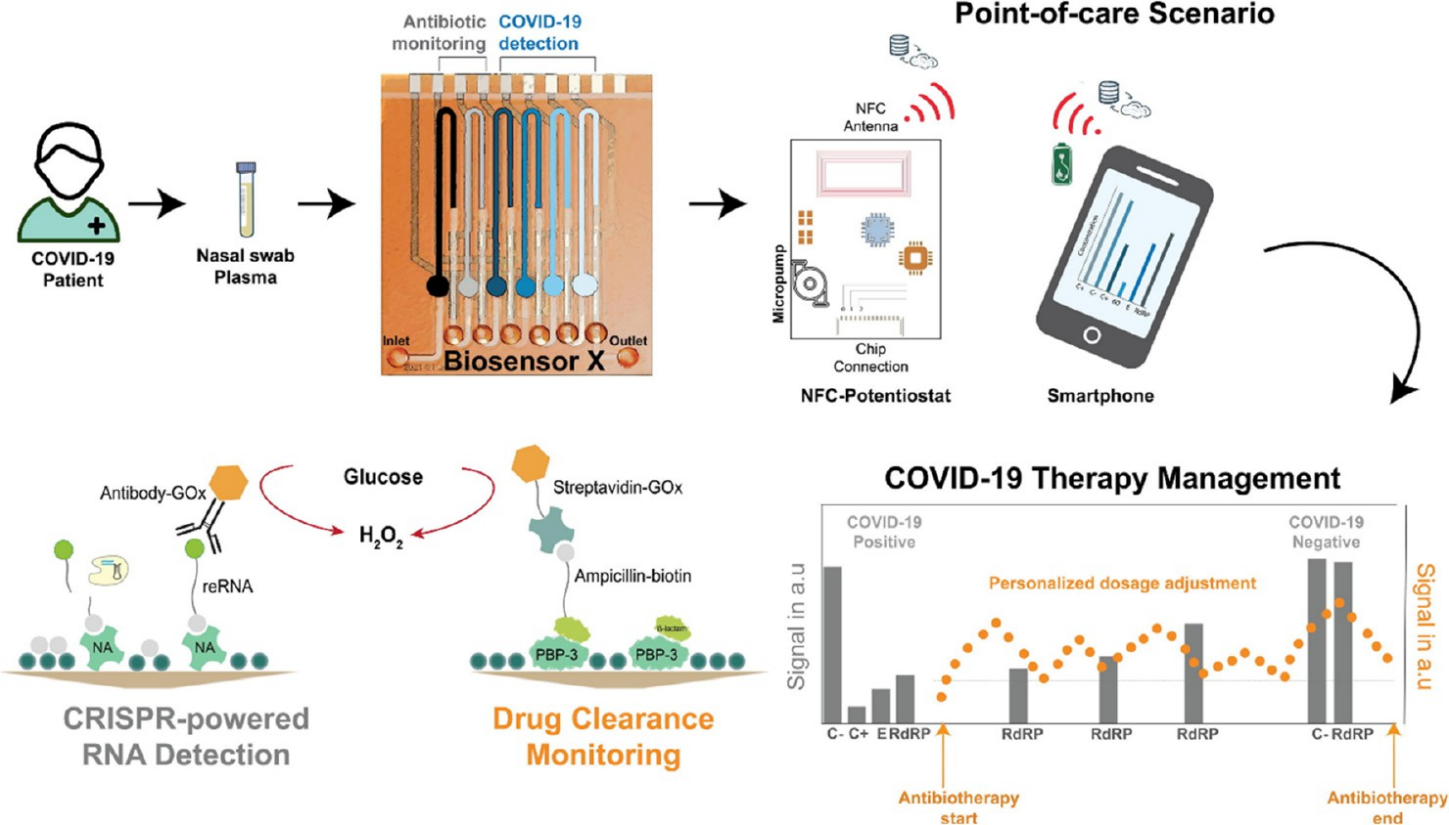
Multiplexed microfluidic bioplatform for the simultaneous determination of viral load and ß-lactam antibiotic in nasal swabs and serum samples from COVID-19-infected patients. Reprinted with permission.^48^ Copyright 2022, Elsevier.

In addition, electrochemical affinity bioplatforms involving aptamer switches and penicillin binding proteins (PBPs, bacterial proteins that bind only the active form of penicillin and other antibiotics of the β-lactam class^105^) have demonstrated to meet the challenging demands of therapeutic drug monitoring (TDM). Remarkable examples are the bioplatforms implemented on gold wires^106^ or needles^107^ for *in vivo* continuous, real-time monitoring of drugs in live rats, and the multiplexed microfluidic bioplatform displayed in Figure 11 for the simultaneous determination of viral load (viral *E* RNA and *RdRP* genes using assays enhanced by CRISPR/Cas, gene editing technology discussed in more detail in the following subsection) and ß-lactam antibiotic in nasal swabs and serum from COVID-19-infected patients, which allowed near real-time assessment of the efficacy of therapy for the treated infection.^48^

Electrochemical affinity biosensors provide also exciting opportunities to advance precision nutrition by interrogating allergen or adulterant indicator targets, regardless of their omics level, origin (plant^108,109^ or animal^110^) and organelle type (nucleus, mitochondrion^91^ or chloroplast) in raw and processed foods, in addition to specific clinical markers (e.g., IgEs and IgG4 selective to the allergenic target) in biofluids.^111^ A representative example is a dual bioelectronic chip for the simultaneous monitoring of vitamins C and D in a 10-μL saliva sample in less than 25 min. This chip is a proof of the versatility of these systems for integrating different detection principles (electrocatalytic and immunoassay) on a single platform and performing rapid simultaneous determinations on small volumes of unprocessed samples.^112^ Another illustrative example is the strategy combining disposable electrochemical bioplatforms and ovoalbumin (OVA)-modified MBs for the reliable determination of OVA-specific IgE and IgG4, which was successfully applied to their analysis in serum samples from egg-allergic children.^111^

### Gene editing and amplification technologies

The use of CRISPR/Cas gene editing technologies,^113,114^ and nucleic acid isothermal amplification strategies,^115−117^ allows the remarkable improvement in sensitivity and selectivity in electrochemical affinity biosensing.

CRISPR/Cas systems consisting of clustered regularly spaced short palindromic repeats (CRISPR) and associated proteins (Cas) are surveillance ribonucleoprotein complexes. They are natural immunization systems in microbes: bacteria contain in their genetic material repetitive sequences of the DNA of viruses that have infected them in the past, allowing bacteria to recognize and defend against such viruses by cleaving their DNA (acting as autovaccines). They combine a unique guide RNA with a Cas system that can be Cas9, deactivated or mutated Cas9 (dCas9, without cleaving activity), Cas12a, and Cas13a.^61^

Simply explained, first, the guide RNA locates the target and hybridizes with it, and then, the Cas protein recognizes the formed structure, which awakens its nuclease activity to cleave the reporter nucleic acid. It is precisely the involvement of these two successive recognition steps that explains the high selectivity of these systems.

It is important to note that in electrochemical affinity biosensors, Cas9 is usually used as the recognition element, whereas Cas12 or Cas13 are often used to amplify the signal due to their multiple turnover trans-cleavage activity.^118^

State-of-the-art electrochemical bioplatforms have been developed by using CRISPR/Cas systems as biorecognition elements for single or multiplexed determination of nucleic acids, or as signal amplifiers to interrogate nucleic acids or other analytes such as proteins, metals, and bacteria.^119−121^ Indeed, the versatility of modern electrochemical platforms and instrumentation involving CRISPR/Cas^122^ technologies allow the determination of markers of different molecular levels and the development of multiplexed electrochemical bioplatforms.

Although their use in electrochemical biodevices is quite recent, the unprecedented possibilities already demonstrated by CRISPR/Cas systems are driving the development of innovative electrochemical biosensing methods with very high sensitivity and selectivity involving, unlike what may seem, simple, fast, and robust protocols. Moreover, CRISPR/Cas technologies have been successfully implemented on paper, integrated into microfluidic devices,^123^ and coupled to other amplification strategies.^124^

In addition, the progress made in coupling electrochemical biosensing with isothermal nucleic acid amplification strategies, specifically helicase-dependent amplification (HDA), loop-mediated isothermal amplification (LAMP), and recombinase polymerase amplification (RPA), which have been successfully implemented even on the electrode surface,^15,116,125^ is considered very important to bring these new platforms to the real practice as PON tests.

**Figure 12:**
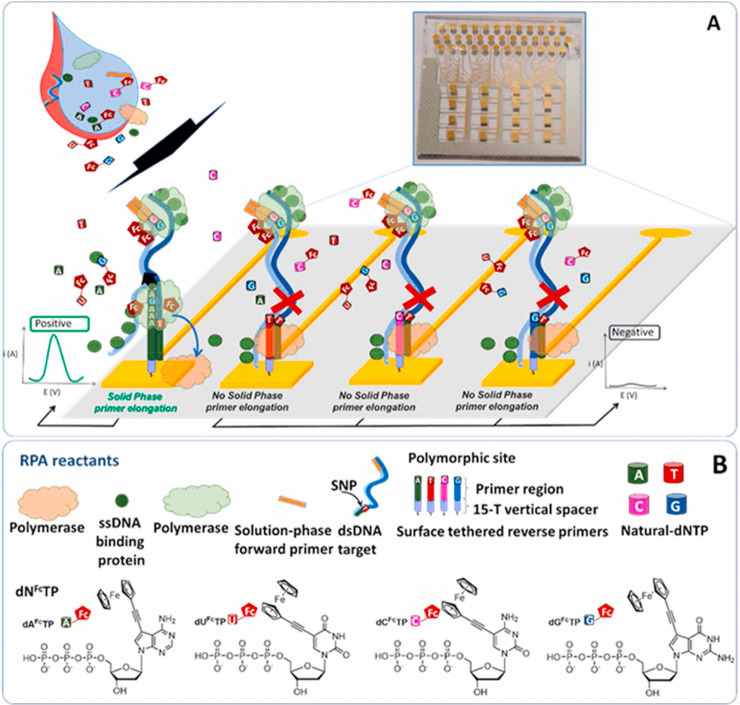
Electrochemical bioplatform for the determination of an SNP in the human genome (hypertrophic cardiomyopathy-associated SNP in the *Myosin Heavy Chain 7* gene) based on an RPA strategy implemented on gold electrodes using Fc-labeled oligonucleotides. Reprinted with permission.^15^ Copyright 2022, Elsevier.

In this context, the development of bioelectroanalytical strategies based on the implementation of surface RPA to develop integrated electrochemical bioplatforms for interrogating bacterial or human genomes^15,116^ can be brought to the scene. As shown in Figure 12, an illustrative example is the determination of a relevant SNP (hypertrophic cardiomyopathy-associated SNP in the *Myosin Heavy Chain 7* gene) in fingerpick blood samples, where RPA was implemented on gold electrodes and Fc-labeled oligonucleotides were used.

### Signal Detection and Processing Techniques

Affinity electroanalytical bioplatforms have cleverly taken advantage of advances in detection techniques, new detection strategies, and processing of electrochemical responses.

Attractive affinity bioplatforms have been also developed involving EIS as a transduction technique, highlighting the need of a thoughtful design of the receptive interface and electrical properties of the interface to achieve both sensitive and reliable biosensing.^126^ Moreover, mixed electrochemical techniques, such as electrochemiluminescence (ECL), have made strong inroads by providing advantages in terms of low background noise, high sensitivity and selectivity, fast response, wide range of detection, low cost, and ease of use.^127,128^

Numerous bioplatforms have been developed by exploiting ratiometric approaches based on the differential measurement and/or the ratio of two electrochemical signals generally with opposite trends and only one of them target-dependent, to ensure the reliability and robustness of the analytical results.^129^

The use of new tools to correct for the baseline drift experienced by in vivo electrochemical responses^106^ and the feasibility of establishing a universal slope for direct calibration-free PON electrochemical immunoassays in serum^130^ brings us a step closer to the development of substantially shorter decentralized electrochemical affinity-based bioassays. Also noteworthy is the recently proposed smart approaches based on orthogonal multipotential redox coding of DNA for targeted electrochemical sequence analysis and genotyping.^131^

**Figure 13:**
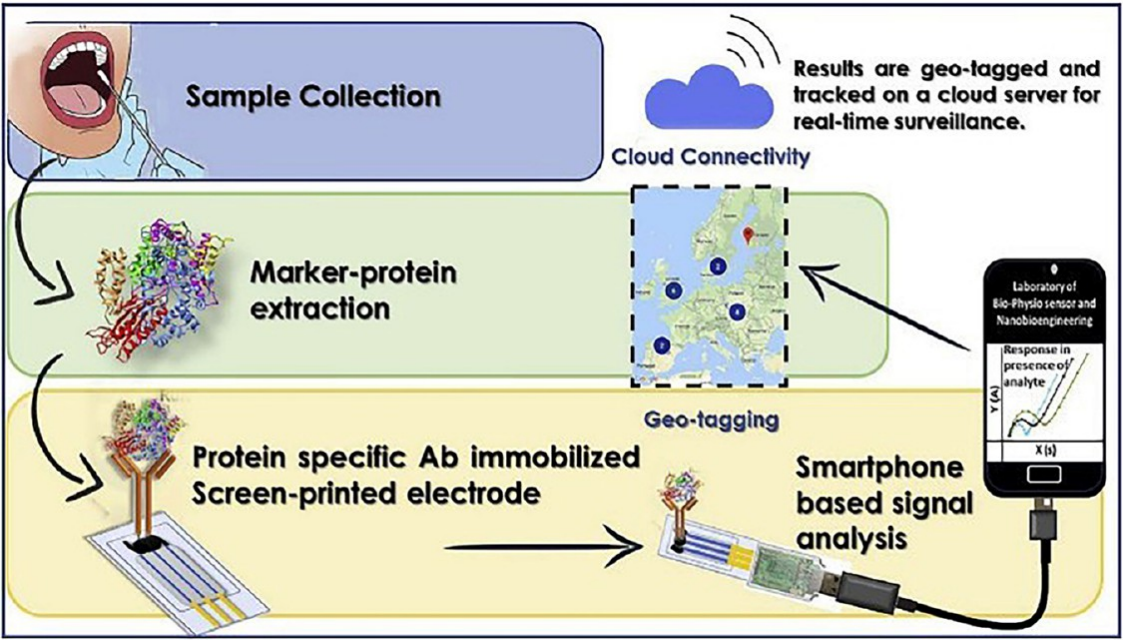
Smartphone assisted electrochemical biosensing for the precise diagnosis of COVID-19. Reprinted with permission.^132^ Copyright 2020, Elsevier.

Advances and developments in smartphone-assisted (Figure 13),^132^ “all-in-one”^133^ and PON electrochemical affinity biosensing platforms are also relentless.^25,134−138^

**Figure 14:**
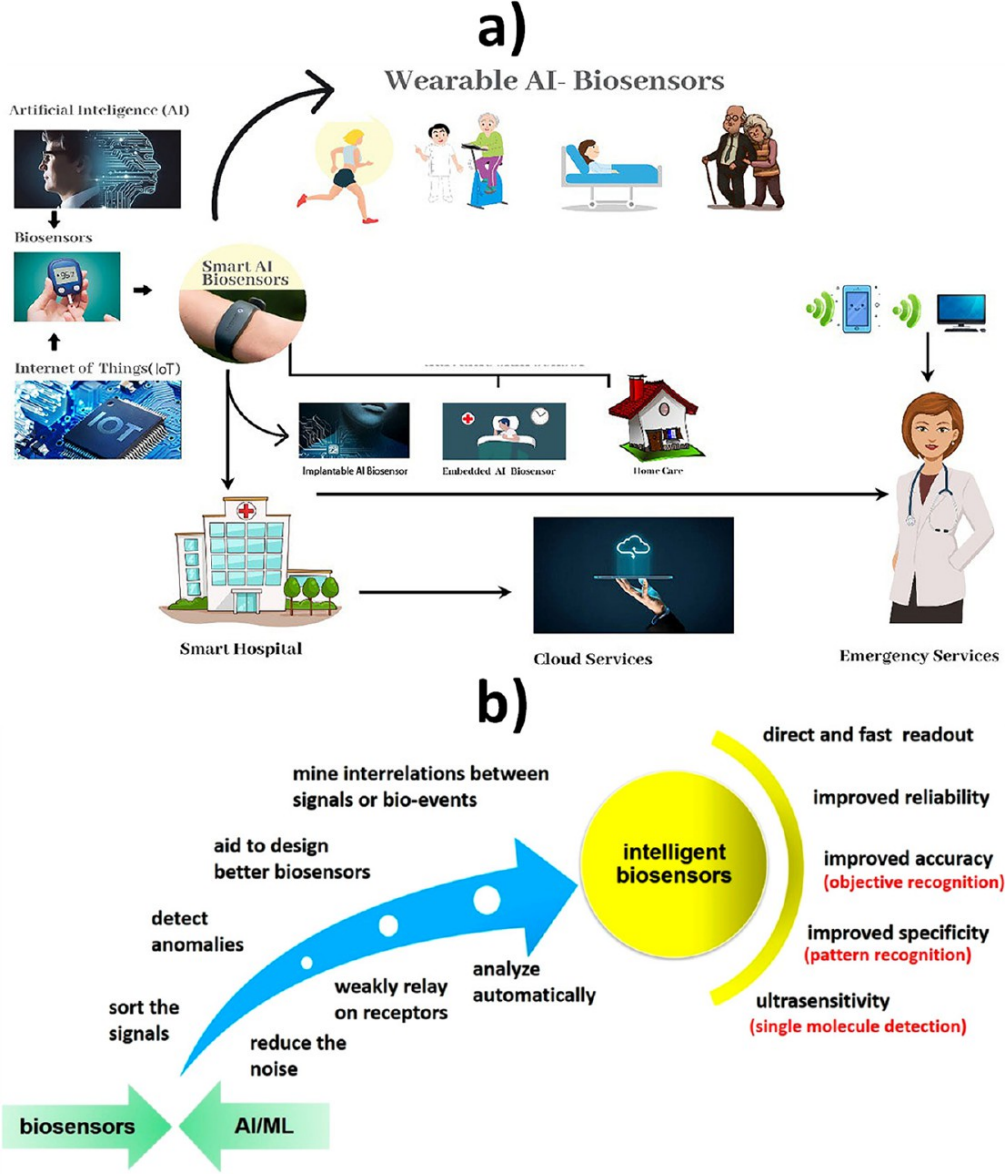
(a) Schematic illustration of AI-IoMT-assisted fifth generation biosensors. (b) Benefits that AI and ML may bring to biosensors. (a) Reproduced with permission.^142^ Copyright 2023, Elsevier. (b) Reprinted with permission.^143^ Copyright 2020, American Chemical Society.

Electrochemical bioplatforms currently have at their disposal the possibility of partnering with Internet of Medical Things (IoMT) (Figure 14a) and artificial intelligence (AI), comprising machine learning (ML) and deep learning (DL) (Figure 14b).^134^ The latter and so-called “fourth dimension” is expected to play a key role in overcoming problems derived from the analysis of real samples, such as electrode fouling, poor signal-to-noise ratio, chemical interferences, and matrix effects, thus improving sensitivity, accuracy, and reliability of the measurements as well as the multiplexing capability.^1,139−141^

## Notes of Interest, Challenges and Exciting Avenues and Horizons Ahead

Since some years ago, we have been astonished by the unstoppable advances of electrochemical affinity biosensing platforms, which never cease to amaze us. These are devices imbued with an open, interdisciplinary, and committed community with an impact that is expected to continue growing at breakneck speed because the impetus they receive is increasingly stronger and from more angles, which makes them increasingly interesting and in demand by multiple sectors.

Not many people would have thought long ago that these biodevices would be able to perform multiomics determinations, implement isothermal amplification of nucleic acids on their surface, be used to discover new markers, to detect SNPs or for genotyping, that they can be ingested, worn, or implanted, and that they would be very promising tools to advance in the study and application of precision medicine, therapy, and precision.

With such a broad and open landscape, it is difficult to predict what we will witness in the coming years. What we do dare to say, looking back a little and being aware of their versatility, adaptability, and open nature, is that they will continue to gain more and more prominence and support in many of the societal demands that will arise.

Moreover, while they indeed have a long and complex road ahead to validate their “academic qualifications” into commercially viable devices,^1,31^ all indications and advances lead us to dream that someday, as is currently the case with our cell phones, we will not be able to live without them and we will consider them as faithful allies of our wellbeing. However, we must be aware that advances are still mainly taking place in research laboratories and that real-world applications are still very limited. Moreover, given all of the challenges to be faced, some of which are discussed below, the market launch of these biodevices is neither close nor easy.

The available sensors are quite idiosyncratic and specific for certain analytes, and the golden dream is to develop biosensing devices that are familiar and comfortable to us, that employ adaptable and universal, reagent-free detection approaches that require no sample washing or handling to easily integrate them into portable devices, i.e., “all-in-one” and “sample-in-answer-out” type devices. Another challenge is related to energy requirements, especially in the case of stand-alone devices (contact lenses, dental implants, etc.) that demand small, lightweight, viable batteries with excellent mechanical flexibility and adaptability.^144^

The robustness and reliability of these sensors must be validated by analyzing a sufficiently representative number of samples in different environments and by different users. Interdisciplinary research and development of these sensors will help to tailor their mechanical, electrochemical and biological properties to specific needs, with a clear path toward their immediate application in daily practice.^144^

Also, on the to-do list is that these devices exhibit true continuous monitoring capabilities as opposed to serial measurements. This is particularly relevant, for example, in the case of inflammation markers. Given the close relationship between prolonged inflammation and certain diseases, such as cancer and cardiovascular disease, their continuous monitoring would provide the opportunity to intervene and prevent disease.

On the other hand, and in addition to all of these practical applicability issues, deeper fundamental studies on mechanistic knowledge, such as affinities, kinetics, diffusion, and rational surface chemistry, are considered essential to develop devices capable of adapting and surviving outside the research laboratory. Evidence of the difficulty of this transition is the very small number of electrochemical biodevices that have bridged this gap compared to the huge number developed, characterized, and applied with promising results in research laboratory settings. There are so many factors involved in the success of this operation that a priori, it is very complex to venture whether the biodevices highlighted in this perspective would be good candidates for it. Moreover, the large efforts and investments from different areas required are currently only prioritized for very particular markers from their wide and ever-growing battery of them. However, if we had to predict the success of market translation among the electrochemical biodevices highlighted in this Perspective, we would bet, because of their unique features and the analytes they target, for the disposable electrochemical chips proposed by Prof. Wang’s group^46,47^ to contribute to the management of diabetes.

The healthcare industry and even the economy are currently being disrupted by the development of AI and IoMT-assisted sensors. Immersed in this disturbing context, future success will depend on the intelligent harnessing of the power of intelligent software and ML methods for the development of ruggedized biodevices. The key to this is to remain aware of the lessons learned from the pandemic, the need to work together to combat unexpected grand challenges, the role of scientific research to ensure the survival of humanity,^142^ and to seek interdisciplinary solutions and compromises between scientists, engineers, users, and entrepreneurs to implement effective ways forward together.^1,145^

We can foresee that electrochemical affinity biosensors will follow in the footsteps of catalytic biosensors, which are a little ahead of them just because they came out earlier, and that in doing so they will redraw new routes and offer different rewards to the former. We can envision that affinity biosensors will be increasingly exploited in wearable,^146^ ingestible and implantable formats and in multiplexed, multiomics, and multimodal devices and that will provide much more information at the molecular level than catalytic biosensors, so essential to bring precision to our lives. They will transform our ability to research and manage nutrition, health, disease, and therapy in an individualized, sustainable, and universally accessible way.

Let us all do our bit to make it happen. The researchers keeping abreast of their advances and of the concurrent exploitable in other disciplines, betting on collaboration to combine them in the best way and having the courage to face increasingly complex and relevant challenges, the producers investing their resources so that these devices can leave their comfort zone and spread their wings outside the research laboratories, and the end users and society giving a vote of confidence to all that these devices can offer, being curious, receptive, and participative in their incursion into daily lives.

## Data Availability

Not applicable.
